# A scoping review of the links between early childhood caries and clean water and sanitation: the Sustainable Development Goal 6

**DOI:** 10.1186/s12903-024-04535-9

**Published:** 2024-07-09

**Authors:** Yasmi O. Crystal, Yuanyuan Laura Luo, Duangporn Duangthip, Maha El Tantawi, Habib Benzian, Robert J. Schroth, Carlos Alberto Feldens, Jorma I. Virtanen, Ola B. Al-Batayneh, Aida Carolina Medina Diaz, Ana Vukovic, Verica Pavlic, Tshepiso Mfolo, Hamideh A. Daryanavard, Balgis O. Gaffar, Anas Shamala, Morẹ́nikẹ́ Oluwátóyìn Foláyan, Early Childhood Caries Advocacy Group (ECCAG)

**Affiliations:** 1 Early Childhood Caries Advocacy Group, Winnipeg, Canada; 2 Department of Pediatric Dentistry, College of Dentistry, New York University, 234 24th Street, New York, NY 10010 USA; 3 College of Dentistry, The Ohio State University, Columbus, OH USA; 4 Department of Pediatric Dentistry and Dental Public Health, Faculty of Dentistry, Alexandria University, Alexandria, Egypt; 5 Department of Epidemiology & Health Promotion and WHO Collaborating Center Quality Improvement & Evidence-Based Dentistry, College of Dentistry, New York University, New York, NY USA; 6 Stellenbosch Institute of Advanced Study, Stellenbosch, South Africa; 7 Department of Preventive Dental Science, Dr. Gerald Niznick College of Dentistry, Departments of Pediatrics and Child Health and Community Health Sciences, Max Rady College of Medicine, Rady Faculty of Health Sciences, University of Manitoba, Winnipeg, Canada; 8 Department of Pediatric Dentistry, Universidade Luterana Do Brasil, Canoas, Brazil; 9 Faculty of Medicine, University of Bergen, Bergen, Norway; 10 Department of Orthodontics, Pediatric and Community Dentistry, College of Dental Medicine, University of Sharjah, Sharjah, United Arab Emirates; 11 Department of Preventive Dentistry, Faculty of Dentistry, Jordan University of Science and Technology, Irbid, Jordan; 12 Pediatric Dentistry and Orthodontics Department, Pediatric Dentistry and Orthodontics Department, Universidad Central de Venezuela, Centro Medico Docente, Caracas, Venezuela; 13 Clinic for Pediatric and Preventive Dentistry, School of Dental Medicine, University of Belgrade, Belgrade, Serbia; 14 Department of Periodontology and Oral Medicine, Medical Faculty University of Banja Luka, 78000 Banja Luka, Bosnia and Herzegovina; 15 Department of Community Dentistry, School of Dentistry, University of Pretoria, Pretoria, South Africa; 16 Dental Department, Dubai Health, Dubai, United Arab Emirates; 17 Department of Preventive Dental Sciences, College of Dentistry - Imam Abdulrahman bin Faisal University, Dammam, Saudi Arabia; 18 Department of Preventive and Biomedical Sciences, Faculty of Dentistry, University of Science & Technology, Aden, Yemen; 19 Department of Child Dental Health, Obafemi Awolowo University, Ile-Ife, Nigeria

**Keywords:** Early Childhood Caries, Clean water and sanitation, Sustainable Development Goal 6, Water fluoridation, Feeding practices, Poverty

## Abstract

**Introduction:**

The United Nation’s Sustainable Development Goal (SDG) 6 calls for universal access to clean water, sanitation and hygiene (WASH), which are crucial elements of health and well-being and fundamental for a life in dignity. Early childhood caries (ECC) is a preventable disease affecting health and quality of life of millions of young children worldwide. This scoping review aims to explore the connection between ECC and access to clean water and sanitation.

**Methods:**

This scoping review, registered on the Open Science Framework and following PRISMA-ScR guidelines, conducted a thorough search in databases (PubMed, Web of Science, Embase, Google Scholar, SciELO) and websites (via Google) in November 2023. The search, without date limitations, targeted studies in English and Spanish linking ECC to SDG6. Exclusions were made for studies solely focusing on ECC without a direct connection to clean water and sanitation. Descriptive statistics summarized the retrieved papers.

**Results:**

The initial search yielded 303 articles. After removing duplicates, 264 articles remained for title and abstract screening after which 244 were excluded and one report was added through citation searching. The 21 remaining articles underwent full text review. There were no studies on a direct association between access to clean water and sanitation and the prevalence of ECC. There were nine studies that showed indirect associations between ECC and access to clean water and sanitation through the links of: water and sanitation access as a marker for poverty (*n* = 1), water consumption as a feeding practice (*n* = 4), and the effectiveness of water fluoridation (*n* = 4). These were used to develop a conceptual model.

**Conclusions:**

While it is conceivable that a direct link exists between ECC and access to clean water and sanitation, the available body of research only offers evidence of indirect associations. The exploration of potential pathways connecting water access to ECC warrants further investigation in future research.

**Supplementary Information:**

The online version contains supplementary material available at 10.1186/s12903-024-04535-9.

## Introduction

Early childhood caries (ECC) is defined as: “the presence of one or more decayed (non-cavitated or cavitated lesions), missing (due to caries), or filled surfaces in any primary tooth of a child under six years old (≤ 71 months)” [1, 2]. A meta-analysis from cross-sectional studies using the World Health Organization’s caries diagnostic criteria showed the global prevalence of ECC was 48% [3], indicating areas of highest prevalence in Oceania (82%) and lowest prevalence in Africa (30%) with differences among regions [4]. When left untreated, ECC can cause pain and infection, with a direct impact on the oral and general health of young children [5]. Severe ECC can impact children’s quality of life affecting the child’s school attendance and performance, as well as having an impact on the family unit [6, 7]. As one of the most common chronic oral diseases among children, ECC is of significant public health importance due to its high global prevalence and impact [8].

Systematic reviews have identified numerous risks factors for ECC, the most relevant include socioeconomic factors (maternal education, family income), behavioral factors (early introduction of sucrose in the diet, frequent consumption of sweet beverages and snacks, non-use of fluoridated toothpaste, poor oral hygiene) and biological factors like high levels of mutans streptococci and lactobacilli, and presence of enamel defects) [9, 10].

Dental caries is a multifactorial disease as is its prevention. Diet, hygiene practices, daily fluoride exposure, selective use of fissure sealants and patient education, are all crucial factors in the prevention of dental caries [11]. In addition, macrolevel factors, like social and economic determinants of health play a major role on disease etiology and its prevention that has to be considered [12]. Visible plaque on the teeth is indicative of poor oral hygiene and is cited as a major risk factor for ECC [13]. Good oral hygiene practices may be dependent on access to water and sanitation just as good hygiene practices may are dependent on access to water and sanitation [14]. However, there is little known about the relationship between ECC and access to safe water, sanitation and hygiene services (WASH) in children younger than 6 years of age. A study on adolescents living in the street suggests that such a link is plausible through an association between good water collection, storage practices and oral hygiene practice [15]. The shortage of access to water and sanitation particularly affects people living in severe poverty such as those living in slums, rural areas and villages [16]. An estimated 26% of the world population is struggling for drinking water and 46% for sanitation [17].

More and more countries are experiencing water stress, and increasing drought and desertification is already worsening these trends [18]. It is projected that at least one in four people will suffer from recurring water shortages by 2050. The global burden of disease and mortality rates could be reduced by about 9.1% and 6.3%, respectively, if rapid success is attained in facilitating access to water, sanitation, and hygiene services [19, 20]. The UN Sustainable Development Goal (SDG) 6, which aims at ensuring access to basic services for water, sanitation and hygiene for all by 2030 [21], provides the main political and programmatic thrust to reduce or eliminate WASH-related health and education impacts. ECC may be a health problem associated with WASH access as the prevalence of ECC is also highest among socially vulnerable children, whose level of poverty predisposes them to live in slums, rural areas and villages where access to water is also a challenge [22, 23]. However, the reality may be more complex, since a number of countries with poor WASH access, notably in sub-Saharan Africa, exhibit low prevalence of ECC [3]. This highlights the need for an understanding of the effect of WASH on ECC prevalence.

Attempting to correlate ECC prevalence by country [4] to availability of WASH services by country [24, 25] is halted by the lack of data from many regions, different times periods of data collection, and the overall heterogeneity of the available information. All these facts emphasize the knowledge gap created by the limited information on the link between SDG 6 and the global burden of ECC. The aim of this scoping review was thus to systematically map and synthesize current evidence on the links between access to water, sanitation and hygiene services and the prevalence of ECC.

## Methods

A scoping literature review was conducted to explore the links between WASH and ECC. A scoping review adopts a broad search strategy while allowing reproducibility, transparency, and reliability on the current state of literature.

### Research question

This review was guided by the question: What is the existing evidence on the links between access to water, sanitation and hygiene services and ECC?

### Protocol and registration

The protocol was registered on the Open Science framework on April 29, 2023, (registration 10.17605/OSF.IO/VZ7U6). This scoping review was conducted in accordance with the Joanna Briggs Institute methodology [26] and reported according to the Preferred Reporting Items for Systematic Reviews and Meta-Analyses Extension for Scoping Reviews (PRISMA-ScR) guidelines [27].

### Articles identification

The initial search was conducted in five electronic databases namely: PubMed, Embase, SciELO, Web of Science, and Google Scholar, as well as gray literature from organizations’ websites in July 2023. The search was performed using the pre-generated query string for the SDG 6 presented in the advanced search function of each database shown in Appendix 1. Search terms were tailored to the specific requirements of each database.

### Eligibility criteria and selection

Inclusion criteria were: 1. Articles published in English and Spanish with no date restrictions, 2. Primary research studies such as clinical trials, case–control, cross-sectional, cohort, and case studies, and those reporting on the link between caries in children under six years of age and access to clean water and sanitation at home, school, and other settings, 3. Systematic Reviews were only included for full-text review if their abstract included some reference to a relationship between water and ECC, 4. Reports from organizations’ websites were only considered if they included some reference to the relationship between water and ECC.

Exclusion criteria were: 1. Review papers were excluded from the full-text review and analysis but were screened for appropriate references.

### Selection of sources of evidence

All identified publications were transferred to reference management software EndNote (X9, Thomson Reuters). Articles that did not meet our inclusion criteria were removed and duplicate articles were removed using the “find duplicates” function. The title and abstract screening of eligible articles were screened by two independent reviewers (YOC, LYL). When there were disparities in findings, this was resolved by consensus between the two reviewers. No authors or institutions were contacted to identify additional sources. All conflicts generated through the screening states between the two reviewers were discussed until consensus was reached.

### Synthesis of results

The information extracted from the publications were the author name, publication year, study location, study design, study sample size and age, study aim, data collection methods, and main findings. The extracted information from each publication was compiled and summarized into Table 1, and a descriptive analysis of the information was conducted. A conceptual model on the link between access to water and sanitation and ECC was developed.

**Table 1:** Nine articles reporting an indirect association with ECC prevalence: access to water as a marker for poverty (*n* = 1), water consumption and feeding practices (*n* = 4), and effectiveness of water fluoridation (*n* = 4)

Author (Publication year)	Location	Design	Sample	Aim	Data collection	Main findings
Inadequate access to water as a marker for poverty						
Folayan et. al 2020 [15]	Global	Ecologi-cal	2007–2017 country level data for 6 Low-Income Countries (LICs) and 45 Middle-Income Countries (MICs) Age: 3–5 years	To assess the relationship between ECC, seven indicators of poverty and the indicator of monetary poverty in LIC and MICs	Country-level data	• The combination of **water and sanitation** with 5 other poverty indicators explained 15% of the variation in the percentage of children with ECC compared to 1% explained by monetary poverty alone • **Water and sanitation** availability had an inverse relationship with ECC prevalence
Feeding practices including water consumption						
Barjatya et. al 2020 [28]	India	Cross-sectional	*n* = 640 Age: 3–5 years	To investigate the association between selected feeding practices and the presence of ECC among children of different socioeconomic status in Indore city	Question-naires & clinical examina-tions	• The overall prevalence of ECC was 64% • Children who were **fed with water** had no ECC, in contrast with children who drank juice (95%), cow or buffalo milk (77%) cold sweetened drink (70%), and infant formula (61%) • ECC scores in children with different bottle contents were found to be significant
Meurman et. al 2011 [29]	Switzer-land	Longitudi-nal	*n* = 366 Age: 18 months -5 years	To identify the early determinants of risk for dental caries to use in its primary prevention	Question-naires & Interview & clinical examina-tions	• The oral health of a child is strongly related to the lifestyle and the oral health habits of the caretakers during the first years • Socioeconomic status of the family seems to be closely related to the oral health habits and oral health • Use of **thirst quenchers other than water** was significantly correlated with the child’s caries increment
Kateeb et. al 2023 [30]	Occupied Palesti-nian Territories	Cross-sectional	*n* = 457 Age: 3–5 years	To determine the prevalence of ECC among preschoolers in a marginalized population and describe the influence of behavioral and social determinants on the development of ECC	Question-naires & clinical examina-tions	• By age 5, 97% had experienced tooth decay, and night feeding habits (putting **things other than water** in the baby bottle at night and/or having children sleep while being breastfed at night) were positively associated with the child developing caries • Late introduction of fluoride toothpaste after age 3, along with the poor feeding habits could explain the elevated level of disease among the sample
Poirier et. al 2022 [31]	Australia	Qualitative study nestled within an Randomized Clinical Trial	*n* = 226 Age: 0–3 years	To collate parental experiences and generate an understanding of facilitators for indigenous childhood oral health	Qualita-tive evaluation of indige-nous parents’ interviews	• Child level facilitators of childhood oral health included **oral health routines** and **regular water consumption** • Many parents identified the importance of water for their child’s health, especially in reducing sugar-sweetened beverage consumption
Water fluoridation						
Masumo et. al 2012 [32]	Uganda, Tanzania	Cross-sectional	*n* = 1221 Age: 6–36 months	To identify possible socio-behavioral correlates of ECC focusing on children and their caretakers living in a high and low fluoride natural water content areas	Question-naires & clinical examina-tions	• Prevalence of ECC was 3.7% in **a naturally occurring high water fluoride** rural area vs. 17.6% in a naturally occurring **low water fluoride** urban area, with both areas reporting children’s sugar consumption to be high
Gomez et. al 2001 [33]	Chile	Cross sectional	*n* = 360 Age: 1–3.5 years	To evaluate the effectiveness of prenatal and postnatal prevention program after the first four years	Calibrated clinical examina-tions	• The preventive dental program was effective in inhibiting caries in pre-school children, even in a population already receiving the benefits of **community water fluoridation**
Moynihan et. al 2019 [34]	UK, Ireland, Canada	Systema tic review	*n* = 13,831	To systematically review published evidence pertaining to the effect of modifiable risk factors on ECC	RCT and cohort studies data	• Evidence from 13 cohort studies showed that providing **access to fluoridated water** is a justified approach to ECC prevention
Marino RJ, and Onetto JE 1995 [35]	Chile	Cross sectional	*n* = 220	To report dental caries prevalence and experience in preschool children in a rural non-fluoridated community and an urban area with optimal water fluoridation	Calibrated dental examina-tions	• Caries experience for the children in urban areas with **optimal water fluoridation** was significantly lower than that for children living in the rural communities with non-fluoridated water

## Results

**Fig. 1:**
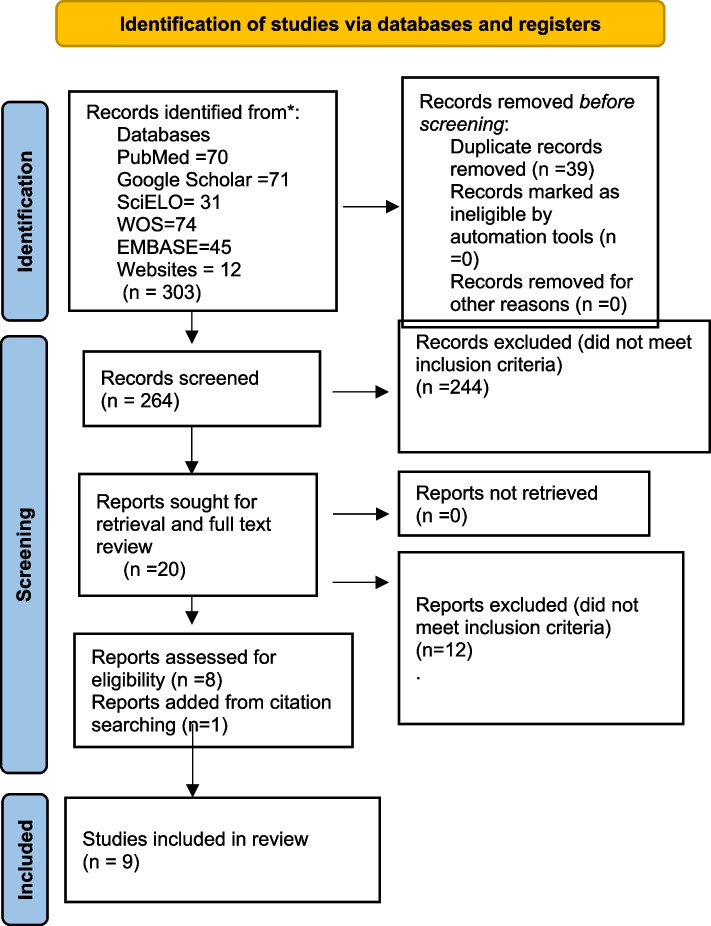
PRISMA flow chart

Figure 1 shows the details of the results of the search of databases, removal of duplicates, screening by title/abstract and full text review. Full text analysis showed that none of these studies described a direct association between access to clean water and sanitation and the prevalence of ECC, but nine studies showed a link between ECC and access to clean water and sanitation-related factors: water as a marker for poverty (*n* = 1) [15], water consumption as a form of feeding practices (*n* = 4) [28–31], and effectiveness of community water fluoridation (*n* = 4) [32–35]. A summary of the 9 included articles and their extracted data is presented in Table 1.

The nine included studies were conducted in Chile [33, 35], Africa namely Uganda/Tanzania [32], Switzerland [29, 31], and India [28], Palestine [30], one study included data from UK, Ireland and Canada [34], and one study included global data by country [15, 22, 36].

The papers were published between 1995 and 2023, the majority (*n* = 4) between 2011 and 2020). Publication details are included in Table 1. The study designs ranged from ecological [15] to cross-sectional [28]; [30, 32, 33, 35], longitudinal [29], qualitative study nestled within a clinical trial [31] and a systematic review [34].

The single study on the link between ECC and access to clean water and sanitation mediated through poverty was an ecological study that indicated that access to water and sanitation had an inverse relationship with the prevalence of ECC [15, 22, 36]. The four studies on the link between ECC and access to clean water and sanitation mediated through feeding practices indicated that the children who consumed alternatives such as sugar-sweetened beverage [28, 31], juice [28], milk [28] and breastmilk throughout the night [30] increased the risk for ECC. In addition, the four studies on the link between ECC and access to clean water and sanitation mediated through fluoridation of water indicated that access to fluoridated water was associated with lower prevalence of ECC [32–35]

**Fig. 2:**
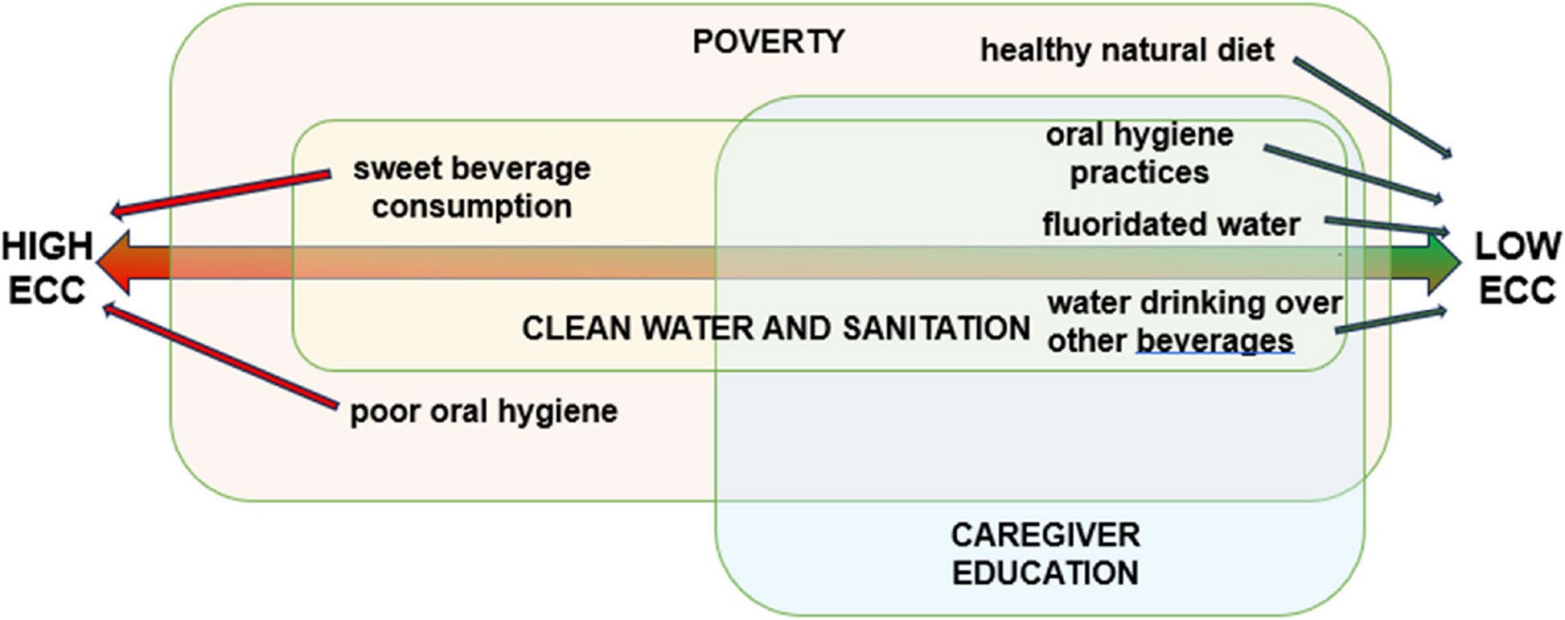
Conceptual model of clean water and sanitation’s associations with the prevalence of ECC

The findings from this scoping review were used to develop a conceptual model illustrating how access to clean water and sanitation may be associated with, as depicted in Fig. 2.

Populations with no access to water and sanitation but on a healthy natural diet, exhibit low ECC. Populations whose diet includes sweet beverage consumption and poor oral hygiene, even with WASH access, have high ECC. Poverty is identified as a dual risk factor affecting both: access to clean water and sanitation [15, 22, 36], and caregiver education [29, 30]. In situations where clean water and sanitation is accessible, the incorporation of water fluoridation serves to reduce the risk of ECC [32, 34, 35]. We postulated that the risk for ECC is further mitigated, even in the presence of fluoridated water, when mothers have access to pre- and postnatal educational interventions [33] that may improve the prioritization of water consumption over other beverages, including prolonged and nighttime breastmilk and other forms of milk [28]; [29–31]. Maternal educational programs would also foster adherence to good oral hygiene practices encompassing effective toothbrushing starting at an early age with the use of fluoridated toothpaste [30]. Even though clean water and sanitation facilitates establishing oral hygiene practices and water drinking over other beverages, these factors seem to be more strongly related to caregiver education and in turn to poverty (being able to afford the tools in addition to having the knowledge to choose healthier habits.

## Discussion

This is the first study exploring the relationship between ECC and access to clean water and sanitation. Although the search results did not identify any articles reporting a direct association between access to clean water and sanitation and the prevalence of ECC, nine articles showing links between access to clean water and ECC in children younger than 6 years of age were identified.

First, we noted that attempting to establish a connection between inadequate access to water, sanitation, and hygiene (WASH) services and ECC may pose challenges due to numerous confounding variables. For instance, people in sub-Saharan Africa have heightened morbidity and mortality due to poor water access [37]. However, the prevalence of ECC in this region is notably low [3, 38]. Also, programs designed to enhance WASH accessibility, aligned with the SDG6, have demonstrated successful outcomes in reducing dental caries prevalence in permanent dentition when implemented in schools [39] Paradoxically, these positive outcomes may not have translated to a similar decline in ECC prevalence as sustained high or increasing prevalence of ECC was observed in these regions in children below age 6 [40]. Notably, successful oral hygiene programs attribute caries prevention outcomes to daily toothbrushing interventions without explicitly mentioning water or clean water. This lack of specificity may explain the absence of studies addressing oral hygiene (toothbrushing) in our findings. The absence of studies defining the importance of water and sanitation on oral-health related hygiene practices and their link with ECC is puzzling because without access to water, personal and oral hygiene are affected. When there is no water in the household, opportunities for hygiene practice are more challenging and often forgone. Without water in schools, hand and oral hygiene programs become difficult to implement, limiting health education and practice [41]. For this reason, SDG 4, which focuses on education, includes a target for universal WASH services in schools.

Second, one of the nine studies that showed a link between access to clean water and sanitation and ECC prevalence suggested that the multifaceted improvements resulting from investments in WASH may have resulted in changes in lifestyles that could potentially increase the risk for ECC in ways unrelated to oral hygiene [15]. This is conceivable as improvements in the standard of living lead to alterations in dietary patterns, including an increase in consumption of unhealthy food which increases the risk for ECC [42, 43]. The study reinforces previous evidence that ECC is strongly linked to poverty [44–46] and poverty is linked to poor WASH access [47]. In addition, WASH access may be a significant socioeconomic marker that may also affect diet, a risk factor for ECC [42]. This risk is mitigated when there is a choice of including water as the main beverage instead of sugar-containing alternatives, which seems to be dependent on the education of parents and caregivers and their (oral) health literacy, and when there is access to fluoridated water, as represented in our conceptual model depicted in Fig. 2. There is reported evidence of different relationships between income and ECC, including inverse linear, positive linear, U-shaped, inverse U-shaped, and no relationship [48] [42, 49–51], This relationship seems to vary based on the local/country/regional trajectory along economic development and the nutrition transition, and rural vs. urban location, primarily mediated by family practices. Epidemiological studies report that minority groups living in poverty in high-income nations with good access to clean water and sanitation, have a higher prevalence of ECC [52]. This has been attributed to low health literacy, limited access to a healthy diet, and reduced access to oral health care. Commercial determinants of oral health may also be at play as low-cost cariogenic foods and beverages are heavily marketed to low-income, ethnic-minority populations. Oral health literacy that comes with education, is key to help vulnerable populations make the right healthy choices. Further research is needed to explore the connections between access to clean water and sanitation and ECC risk. Such studies can help understand the economic and dietary changes that co-occur with successful WASH interventions and may be related to ECC risk.

Third, access to naturally fluoridated water and community water fluoridation reduces the risk of ECC [32–35]. Access to optimally fluoridated water is less likely when access to centrally-managed piped water systems is poor. Identified studies on the effect of water fluoridation on children aged 6 and under indicated that exposure to fluoridated water reduces the risk for and severity of ECC [32–35]. Community water fluoridation as a public health measure is considered a safe, effective, and socially equitable means of achieving community-wide protection against dental caries [53, 54]. regardless of age, education, income level, or access to routine dental care [55]. For these reasons, there is a call for the prevention of dental caries through access to optimum fluoridated water [8]. Achieving the goals of SDG6 would facilitate the promotion of this public health measure. However, some studies highlight that the impact of fluoride on the risk of caries may be obscured by the impact of other behavioral variables such as excessive sugar consumption, the presence of plaque or length of breastfeeding [32, 56], underscoring the overriding impact of education. In addition, the continued call for water fluoridation is complex as there are concerns surrounding both the ethics [57], and the efficacy of systemic fluoride for caries prevention [58]. To further complicate this topic, there is strong evidence that areas with poor access to clean water and sanitation and therefore no water fluoridation, have low ECC prevalence [3] and countries without community water fluoridation can achieve impressive caries reductions through programs that offer continuous oral health promotion programs from birth [59, 60].

Fourth, we also found studies suggesting that feeding practices that included water rather than sweetened beverage, were protective against ECC [28–31], in agreement with evidence on the protective effect of consuming water versus sweetened beverages [61]. A study also reported that children who transition directly from breastfeeding to water had no increased risk of ECC [31]. This finding does not have such a strong connection to clean water availability as much as it has to the access to information by the child’s caregiver to know to choose between drinking water vs. drinking other beverages [28, 29, 31]. This introduces yet another layer of complexity on the link between access to water and ECC, as education may mediate this link.

An additional related fact is that access to unsafe water and poor sanitation increases the risk of children to diarrhea [62] and malnutrition [63], leading to anemia [36, 64] and all are risk factors for defective enamel formation [65], a known risk factor for ECC [66]. Carefully designed studies are therefore needed to assess the impact of access to clean water and sanitation on the risk for ECC, and to understand the pathways for the impact.

A limitation of this study is that we only searched and included studies published in English or Spanish which were the languages the literature search team were proficient in. There may have been publications in other languages that were not included which potentially underestimates the literature on the studied association. However, we covered the bulk of the literature which is usually produced in English. Another limitation of this specific topic is that databases of clean water and sanitation availability as well as those that report ECC prevalence by country, are not comprehensive. There is missing data from many regions, heterogeneity on the periods of data collection, and little data reported on children younger than school age. Since the SDGs were adopted in 2015, research conducted within the context of SDGs in relation to health aspects might still be lacking, which may explain the limited findings in our search. Limitations also arose due to variations in the definition and diagnostic criteria of ECC [44] which we addressed by expanding the search terms. Another limitation is that the literature review was limited to associations between WASH and ECC, thereby possibly not capturing studies involving older age groups where associations may be different than in the age groups affected by ECC [22, 39, 67].

The strengths of our study lie in the fact that we registered and followed a strict protocol adhering to recognized guidelines, and the search was conducted with the help of an experienced librarian at a major university. After the data was extracted by a core group, a team of 17 authors each from different countries, ethnic backgrounds and work settings, critically appraised and discussed the findings, bringing in diverse perspectives.

The findings from this scoping review reveal a paradox in relation to SDG 6, which aims for universal access to clean water and sanitation. Despite the goal’s success in reducing the burden of water-related systemic diseases, regions with poor water access, notably sub-Saharan Africa, exhibit low prevalence of ECC. In addition, WASH programs, while successful in improving permanent dentition outcomes, do not always reach the children that at younger ages are not yet in school settings that would allow them to benefit from such programs. Furthermore, access to clean water and sanitation serves as a socioeconomic marker influencing ECC risk, and it affects the likelihood of accessing fluoridated water. Education is suggested to mediate the link between water access and ECC, adding complexity and emphasizing its role in achieving SDG 6 objectives. The multifaceted nature of the ECC-water link aligns with SDG 6's comprehensive approach, necessitating carefully designed studies to assess the impact of water and sanitation access on ECC, considering the interplay of health, education, and socioeconomic factors. Studies are also needed to clearly delineate the links between water availability and hygiene practices including toothbrushing with fluoride toothpaste in young children.

## Conclusions

In conclusion, though it is plausible for there to be a direct association between ECC and access to clean water and sanitation, the current body of research-based evidence only provides evidence on indirect associations. The conceptual framework developed from the body of evidence needs to be explored further as more research is conducted in these areas. The study findings contribute to a nuanced understanding of the challenges in directly associating water access with ECC prevalence. The complexities identified underscore the importance of holistic strategies that address various determinants, aligning with the comprehensive goals of SDG 6. Further research and tailored interventions are essential for achieving sustainable improvements in water, sanitation, and hygiene which will lead to better oral health outcomes.

### Supplementary Information

Supplementary Material 1.

## Data Availability

All data is provided within the manuscript and supplementary information files.

## Abbreviations

ECC: Early childhood caries
SDG 6: Sustainable Development Goal #6
WASH: Water access, Sanitation and Hygiene
UN: United Nations
LIC: Low income countries
MIC: Middle income countries
