# Supplementary material for: A scoping review of the links between early childhood caries and clean water and sanitation: the Sustainable Development Goal 6
Source: BMC Oral Health. 2024 Jul 9;24:769. doi: 10.1186/s12903-024-04535-9 (PMC11234638; doi:10.1186/s12903-024-04535-9)
Supplement: Supplementary file 1 — Supplementary Material 1. [file 12903_2024_4535_MOESM1_ESM.docx]

**Appendix 1**

1. Search Terms: **Embase search**: ((Early childhood caries or ECC) and (water or water insecurity or clean water or sanitation) and prevention).mp. [mp=title, abstract, heading word, drug trade name, original title, device manufacturer, drug manufacturer, device trade name, keyword heading word, floating subheading word, candidate term word] (n=45)
2. **PubMed search**: ((("early childhood caries"[All Fields] OR "ECC"[All Fields]) AND ("water"[MeSH Terms] OR "water"[All Fields] OR ("sanitation"[MeSH Terms] OR "sanitation"[All Fields] OR "sanitizers"[All Fields]))) AND ("prevent"[All Fields] OR "preventability"[All Fields] OR "preventable"[All Fields] OR "preventative"[All Fields] OR "preventatively"[All Fields] OR "preventatives"[All Fields] OR "prevented"[All Fields] OR "preventing"[All Fields] OR "prevention and control"[MeSH Subheading] OR "prevention and control"[All Fields] OR "prevention"[All Fields] OR "preventions"[All Fields] OR "preventive"[All Fields] OR "preventively"[All Fields] OR "preventives"[All Fields] OR "prevents"[All Fields])) AND (english[Filter]) (n=66)
3. **Web of Science Search**: LA=(English) AND ALL=(early childhood caries or dental caries ) NOT ALL=(fluoride or fluoridation or water fluoridation) AND ALL=(clean water or water insecurity or sanitation) (n=78)
4. **SciELO Search:** early childhood caries OR ECC AND water OR clean water OR sanitation (n=31)
5. **Google Scholar Search:** "early childhood caries" and ("clean water" or water) and (sanitation or hygiene) and prevention (setting: English) (n=68)
